# Retrospective Analysis of Totally Implantable Venous Access Ports which was Performed using the Patient’s Height as a Guide, and the Effect of Catheter Tip Position on Complications

**DOI:** 10.4274/TJAR.2026.252308

**Published:** 2026-04-15

**Authors:** Hakkıcan Akpolat, Serdar Demirgan, Sezen Kumaş Solak, Emine Sönmez, Rasim Onur Karaoğlu, Ayşin Selcan

**Affiliations:** 1 University of Health Sciences Türkiye, Bağcılar Training and Research Hospital, Clinic of Anaesthesiology and Reanimation, İstanbul, Türkiye; 2 İstanbul University, Institute of Graduate Studies in Sciences, Department of Molecular Biology and Genetics, İstanbul, Türkiye

**Keywords:** Catheter tip positioning, cavoatrial junction, chemotherapy, insertion technique, totally implantable venous access port

## Abstract

**Objective:**

Totally implantable venous access ports (TIVAP) provide safe and com-fortable venous access for chemotherapy. This study evaluates the reliability of Lum’s measurement technique for central venous catheter tip positioning and its impact on complications.

**Methods:**

Clinical and radiologic data of 297 patients under-going TIVAP implantation were analyzed. The primary endpoint was optimal catheter tip positioning (within 2 cm above to 1 cm below the cavoatrial junction) and its effect on complications. Secondary endpoints included the impact of catheterization site and tip position relative to the carina.

**Results:**

Among 297 patients, 59.9% had catheter tips in the target zone, and 93.9% were below the carina. Target zone positioning did not significantly affect catheter occlusion or thromboembolism (*P*=0.066, *P*=0.773). However, thromboembolism (1/18; 5.6% vs. 1/279; 0.4%, *P*=0.009) and catheter occlusion (2/18; 11.1% vs. 3/279; 1.1%,* P*=0.001) were more frequent when the tip was above the carina. Patients with tips in the target zone and below the carina had similar complication rates (*P*=0.565,* P*=0.748, *P*=0.644). Catheterisation was performed via the internal jugular vein (IJV) or subclavian vein (SCV). Target zone positioning was more frequent with IJV catheterization (*P*=0.047), while catheter occlusion was higher with SCV access (*P*=0.024).

**Conclusion:**

Positioning the catheter tip below the carina and preferring IJV as the first-choice catheterization site may reduce complications

**Main Points**

• Lum's height-based measurement technique achieved correct catheter tip positioning within the predefined cavoatrial junction (CAJ) target zone in approximately 60% of cases, indicating moderate predictive accuracy.

• Catheter tip placement above the carina was significantly associated with higher rates of thromboembolism and catheter occlusion.

• Positioning the catheter tip below the carina appeared to be a more clinically relevant determinant of complication risk than strict adherence to the predefined CAJ target zone.

• The internal jugular vein approach was associated with more optimal tip positioning and fewer catheter occlusions compared with the subclavian vein approach.

## Introduction

Totally implantable venous access ports (TIVAPs) have become essential components of modern oncology practice, providing reliable, long-term venous access for the administration of chemotherapy and other repeated intravenous treatments. Despite their widespread use and the convenience they offer, several procedure- and device-related complications may occur during or after implantation. One of the most important issues is incorrect catheter tip positioning, which is associated with both early and late complications, including venous thrombosis, catheter malfunction, vascular injury, perforation, arrhythmias, valvular trauma, and myocardial damage.^1, 2^

Determining the appropriate catheter length and ensuring accurate tip placement within the superior vena cava (SVC) are therefore crucial for minimizing catheter-related complications. Although the ideal location of the central venous catheter (CVC) tip remains controversial,^3, 4^ most experts agree that the lower segment of the SVC and the cavoatrial junction (CAJ) represent the safest target positions.^5, 6, 7^ Previous studies have suggested that the CAJ can be approximated on a posteroanterior chest radiograph as a point.^2^ Four vertebral body units (VBUs) inferior to the carina.^4^

TIVAPs are commonly indicated for repeated chemotherapy administration, long-term parenteral nutrition, extended antimicrobial therapy, and for patients with limited peripheral venous access. Complications associated with these devices vary over time; early complications include pneumothorax, arterial puncture, and superficial infection, whereas late complications may involve thrombosis, catheter occlusion, malposition, arrhythmias, or myocardial perforation.^1, 2^ Therefore, ensuring that the catheter tip is positioned in the lower SVC, close to the CAJ, is considered fundamental to safe device placement, although numerical definitions of “optimal” tip position differ among guidelines and published studies.^3, 4^

Several techniques are currently used to estimate the optimal catheter length and verify the catheter tip location in clinical practice, including fluoroscopy-guided placement, intracardiac electrocardiography (ECG), anthropometric measurements, surface landmark-based methods, and the catheter-length measurement technique introduced by Lum’s measurement technique (LUM).^8, 9, 10^ LUM’s method has gained attention because it is simple, does not require specialized equipment, and can be applied in various clinical settings without extensive training.

The present study retrospectively evaluated the accuracy of LUM’s height-based catheter length measurement technique in predicting the correct catheter-tip location using chest X-ray landmarks. In addition, the relationship between catheter tip position and associated complications was analyzed. Patient satisfaction with TIVAP implantation was also assessed.

## Methods

### Patient Selection

This retrospective study was conducted after ethical approval by the University of Health Sciences Türkiye, Bağcılar Training and Research Hospital Ethics Committee (approval no.: 2023/10/09/065, date: 27.10.2023). Medical records of patients who underwent TIVAP insertion via the right internal jugular vein (IJV) or right subclavian vein (SCV) between January 2021 and January 2023 were reviewed. Of 314 eligible patients, 297 were included in the final analysis. All patients had a confirmed diagnosis of malignant neoplasms and required a totally implantable venous access device for chemotherapy. Written informed consent was obtained from all patients prior to the procedure.

Inclusion criteria were as follows: age between 18 and 75 years; ability to provide informed consent; availability of a postoperative chest X-ray; no clinical requirement for conventional CVC placement; and absence of contraindications to right-sided venous access. Patients were excluded if they were pregnant or had any of the following: a pacemaker, previous central venous access procedures, anatomical alterations due to thoracic surgery, mediastinal invasion by lung cancer, significant pulmonary disease, spinal deformities, or prior spinal surgery.

**Figure 1:**
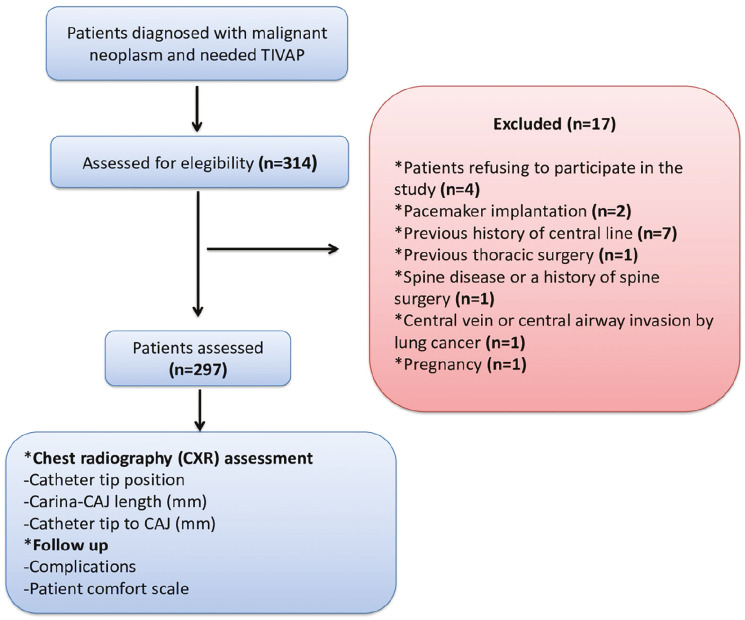
Study flow diagram. TIVAP, totally implantable venous access ports; CAJ, cavoatrial junction; CXR, chest radiography.

Demographic and clinical data, including age, sex, height, weight, body mass index (BMI), American Society of Anesthesiologists (ASA) classification, comorbidities, malignancy type, duration of catheterization, insertion site, and catheter tip location, were recorded. All patients were followed for at least six months to monitor early postoperative events, including pneumothorax, accidental arterial puncture, local infection, catheter-associated sepsis, occlusion, tip migration, venous thrombosis, and mortality. Procedural variables such as arrhythmias, puncture site, number of attempts, and ultrasound guidance were also documented. A flow diagram of patient selection is presented in Figure 1.

### TIVAP Implantation Technique

All TIVAP insertions were performed by experienced anaesthesiologists in a fully equipped operating room under sterile conditions. Standard monitoring, including ECG, non-invasive blood pressure, and pulse oximetry, was applied to all patients throughout the procedure.

Patients received sedoanalgesia and were continuously monitored to ensure comfort and hemodynamic stability. The procedure was carried out under local anaesthesia  with 2% lidocaine infiltration at the puncture and port pocket sites. No general anaesthesia or deep sedation was administered, and all patients remained conscious and cooperative.

Before the procedure, a detailed medical history was obtained and routine preoperative investigations, including coagulation parameters, complete blood count, ECG, and vascular ultrasonography, were completed. The PORT-A-CATH system (Polysite 4000, Vygon, France) was used in all cases.

For right IJV catheterization, patients were positioned in a 10° Trendelenburg position with their heads gently rotated to the left. For right SCV access, the same Trendelenburg tilt was applied, and the arm was kept adducted to minimize catheter malalignment.^11^

After sterile preparation of the puncture site, venous puncture was performed using an 18-gauge needle, with or without ultrasound guidance (Esaote MyLab Five, Genoa, Italy), depending on operator preference and equipment availability. Catheter placement followed the Seldinger technique. Ultrasound guidance was used whenever available, but was not mandatory due to logistical constraints.

The traditional Seldinger method—advancing beyond the posterior vessel wall and withdrawing until a return of blood—was applied.

A straight subcutaneous tunnel was created to prevent catheter kinking, and catheter mobility was confirmed before port attachment. A 3-cm incision parallel to the clavicle was made using a no. 15 scalpel blade. Blunt dissection was used to create an adequate port pocket, and the chamber was positioned subcutaneously below the puncture site.

Catheter length was determined using LUM’s height-based measurement technique. For right IJV access, the formula used was height (cm)/10+2 cm; for right SCV access, it was height (cm)/10+1 cm.

Although this method is simple and does not require fluoroscopy or intracardiac ECG, it does not account for individual anatomical variability, which may affect its predictive accuracy and may explain why some catheters were positioned outside the ideal target zone.

A postoperative chest radiograph was obtained to evaluate for early complications and confirm the catheter tip position.

### Patient Comfort Assessment

Patient comfort was evaluated using the numeric rating scale (NRS), ranging from 0 (no discomfort) to 10 (worst imaginable discomfort). The assessment was performed on postoperative day 7 by a nurse blinded to catheter tip position. The NRS reflected overall procedural comfort, including pain, anxiety, and procedural tolerance. The NRS is a validated and widely used tool for postoperative comfort assessment.^12^

### Radiological Measurements

All postoperative chest radiographs were reviewed by board-certified radiologists. Measurements included the distances between the catheter tip and the CAJ (in millimeters) and between the carina and the CAJ. The position of the catheter tip relative to the carina (superior or inferior) was also recorded. The predefined “target zone” extended from 2 cm above to 1 cm below the CAJ.^13^

**Figure 2:**
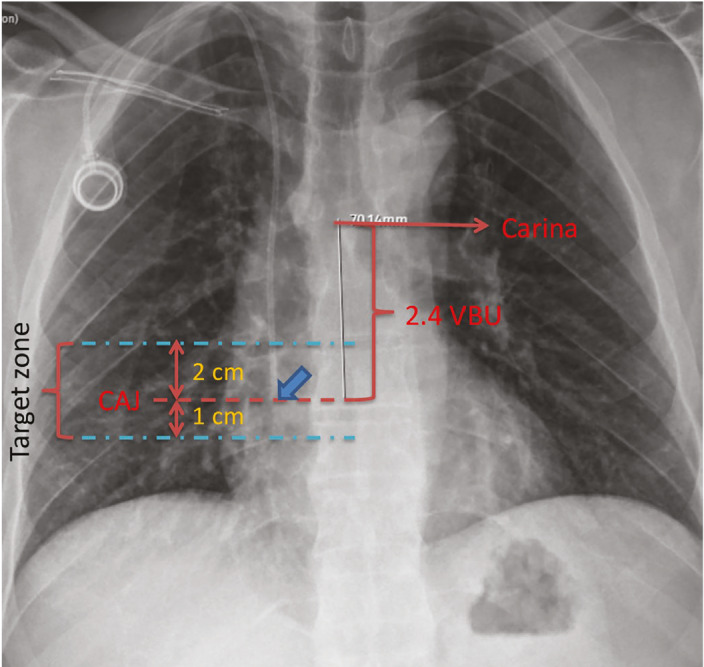
Measurements on chest radiography used to determine catheter tip position. The figure shows a totally implantable venous access port catheter tip positioned exactly at the CAJ. Blue arrow indicates position of catheter tip. CAJ, cavoatrial junction; VBU, vertebral body units.

A VBU was defined as the vertical distance from the inferior endplate of one vertebra to that of the adjacent lower vertebra, including the intervertebral disc space. The carina was identified radiographically as the point of bronchial bifurcation. The CAJ was assumed to be located 2.4 VBUs below the carina (Figure 2).^3^

### Outcomes

The primary outcome was the relationship between catheter tip position relative to the carina and catheter- or procedure-related complications.

Secondary outcomes included the association between complications and catheter tip placement within the predefined CAJ target zone (from 2 cm above to 1 cm below the CAJ), as well as the effects of catheterization site and ultrasound guidance on complication rates.

### Statistical Analysis

Statistical analysis was performed using GraphPad Prism version 7 (La Jolla, CA, USA). Normality was assessed with the Shapiro-Wilk test. Continuous variables were expressed as means ± standard deviations or medians with interquartile ranges, as appropriate. Categorical variables were presented as counts and percentages.

The Mann-Whitney U test was used for non-normally distributed continuous variables. Categorical data were analyzed using the chi-square test. Correlations were evaluated using Spearman’s correlation coefficient.

A *P* value < 0.05 was considered statistically significant. Very small *P *values were reported as *P*  < 0.0001. No formal corretion for multiple comparisons was applied because subgroup analyses were considered exploratory.

## Results

### Patient Characteristics

Of the initial cohort of 314 individuals, 297 patients were included in the final analysis (Figure 1). Of these, 154 (51.8%) were women and 143 (48.1%) were men. The median age was 61 years (range: 23-82). The mean height, weight, and BMI were 165.2±8.3 cm, 68.9±12.7 kg, and 25.3±4.8 kg/m^2^, respectively. Hypertension was the most common comorbidity (n = 89), while colorectal malignancy was the most frequent cancer diagnosis (n = 131). Detailed demographic and clinical characteristics are presented in Table 1.

### TIVAP Insertion Characteristics and Procedural Safety

The mean duration of catheter use was 234.5 days, and patients received a mean of 12 chemotherapy treatments (range: 1-50). Ultrasound guidance was used in 55% of venous punctures, and two-thirds of all procedures were completed with a single attempt.

In the IJV group, one attempt was sufficient in 70.3% of cases compared with 63.1% in the SCV group; however, this difference was not statistically significant (*P*=0.115). Arterial puncture occurred at similar rates in IJV (8.8%) and SCV (7.4%) catheterizations (*P*=0.658). Pneumothorax occurred in 1.3% of patients in both groups (*P*=0.995). Transient arrhythmias were observed in 84.5% of procedures. In 3.03% of cases, the access site was changed due to failed attempts.

The overall complication rate was 11%; the specific complications listed are not mutually exclusive: local infection (0.34%), thromboembolism (0.67%), catheter occlusion (1.7%), pneumothorax (1.7%), and arterial punçture (8.1%). None of the arterial puncture cases resulted in pseudoaneurysm, significant hematoma, hemothorax, hemomediastinum, or inadvertent arterial cannulation.

**Figure 3:**
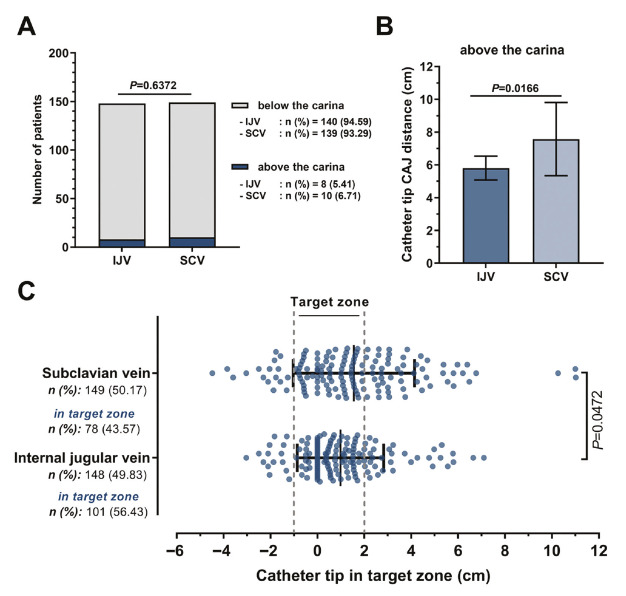
Comparison of IJV and SCV approaches according to catheter tip position. (A) The distribution of patients in terms of the position of the catheter tip relative to the carina was similar between groups (*P*=0.637). (B) Catheter tip-CAJ distance of the patients with catheter tip located above the carina was higher in patients with SCV catheter (*P*=0.016). (C) The number of patients with catheter tips in the target zone was higher in IJV approach (*P*=0.047). CAJ, cavoatrial junction; IJV, internal jugular vein; SCV, subclavian vein.

TIVAPs were inserted via the right IJV in 148 patients and via the right SCV in 149 patients. Significantly more IJV catheters were positioned within the predefined target zone compared with SCV catheters (*P*=0.047). Catheter tips located above the carina showed a greater tip-to-CAJ distance for SCV insertions than for IJV insertions (*P*=0.016). However, the proportion of catheters positioned above versus below the carina did not differ between the two access sites (*P*=0.637) (Figure 3).

Ultrasound guidance was used significantly more often for IJV access (97.3%) than for SCV access (9.6%) (*P* < 0.0001). Catheter occlusion occurred more frequently in SCV catheterizations (3.4%) than in IJV placements (0%) (*P*=0.024). The relationship between insertion site, complications, and patient comfort is summarized in Table 2.

### Imaging Findings and Accuracy of Catheter Tip Placement

On chest radiographs, the mean carin-CAJ distance was 57.05±5.86 mm. The mean distance from the catheter tip to the CAJ was 12.71±22.53 mm (range: -44.80 to +110.20 mm). Optimal catheter tip positioning was achieved in 59.9% of evaluable patients (178/279). Most catheter tips (93.9%) were located inferior to the carina (Table 3).

Thromboembolism occurred in only 0.36% (1/279) of patients whose catheter tips were below the carina, but increased to 5.6% (1/18) when tips were positioned above the carina (*P*=0.009). Catheter occlusion was also more frequent among patients with catheter tips positioned above the carina (11.1% vs. 1.1%, *P*=0.001) (Table 4).

Complication rates did not differ significantly between patients with catheter tips inside the target zone and those outside it (occlusion *P*=0.066; thromboembolism *P*=0.773). Catheter longevity was also similar between groups (below the carina: median 200 days vs. above the carina: median 180 days; *P*=0.124).

When patients with catheter tips either within the target zone or below the carina were compared, similar rates of occlusion and thromboembolism were observed. These findings emphasize the importance of achieving catheter tip placement below the carina—particularly when using the IJV approach—to minimize complication risk.

### Correlation Analysis

**Figure 4:**
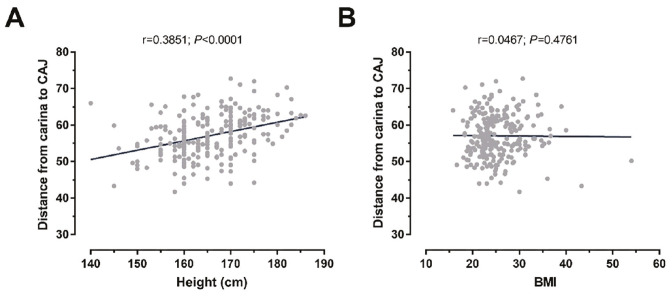
Correlation analyses. (A) Distance between carina and CAJ positively correlated with patients’ height (r = 0.3851; *P* < 0.0001). (B) A significant but weak correlation was found between age and mortality (r = 0.2776; *P* < 0.0001). CAJ, cavoatrial junction; BMI, body mass index.

A moderate positive correlation was observed between patient height and the carina-CAJ distance (r = 0.3851, *P *< 0.0001). No significant association was found between BMI and this measurement (r = 0.0467, *P*=0.4761) (Figure 4).

Mortality showed a weak but statistically significant correlation with age (r = 0.2776, *P* < 0.0001). No significant correlation was observed between mortality and ASA classification or the presence of metastatic disease.

## Discussion

In this retrospective analysis, we examined the reliability of LUM’s height-based CVC measurement method for predicting the final tip position of TIVAP catheters placed via the right internal jugular or SCV and assessed whether tip location influenced complication rates in 297 patients. Our data indicate that the technique achieved accurate positioning in approximately 60% of cases on postoperative chest radiographs, suggesting only moderate clinical effectiveness. Importantly, most catheters (93.9%) were located inferior to the carina, and these patients experienced fewer complications. In contrast, tip placement above the carina was significantly associated with higher rates of thromboembolism and catheter occlusion.

This observation supports existing physiological explanations that catheter tips situated below the carina are exposed to more stable blood flow patterns and to less mechanical irritation of the vessel wall, thereby reducing adverse events.^4^ Additionally, our results demonstrated that the IJV approach yielded higher rates of correct tip placement and fewer catheter occlusions compared with the SCV approach.

LUM’s formula, which determines catheter length according to patient height and puncture site, is appealing because it requires no specialized equipment and is adaptable to many clinical scenarios. Nevertheless, inaccurate tip positioning can lead to several complications, including thrombosis, malfunction, or infection, particularly when the catheter terminates too proximally within the SVC.^3^ Although most of the literature identifies the ideal termination point as the lower segment of the SVC near the CAJ,^14, 15, 16^ European recommendations also regard a position up to 2 cm below the CAJ as clinically acceptable.^17^ Although only 59.9% of catheters were positioned within the predefined target zone, placement within this zone did not independently predict complications in this study. Instead, catheter position relative to the carina was the dominant determinant of clinical outcomes.

When patients whose catheter tips were either in the target zone or positioned below the carina were compared, complication rates were similar. These findings suggest that achieving subcarinal tip placement may be sufficient for clinical safety and may be consistent with international guideline recommendations.

The right internal jugular and SCVs are the most frequently used access sites for CVC.^18^ Although both approaches were used equally in our cohort, the SCV route was associated with higher rates of thromboembolism and catheter occlusion. This difference appeared to be influenced primarily by the final tip position rather than by the access site itself. Catheters inserted via the IJV were more frequently positioned within the optimal zone. These findings are consistent with previous reports, including a meta-analysis of 3,905 patients demonstrating fewer mechanical complications with IJV access than with SCV access.^19^

Cardiac arrhythmias are well-documented events during CVC placement.^20^ In our cohort, transient arrhythmias occurred in approximately 85% of procedures. Importantly, all arrhythmias were self-limiting and clinically insignificant, and no hemodynamic instability was observed. Patient comfort levels, as reflected by NRS scores, remained acceptable.

The arterial puncture rate in our study (approximately 9%) was higher than that reported in some earlier studies.^2, 12^ This may reflect operator experience during the study period, use of static rather than continuous real-time ultrasound guidance in some cases, and anatomical factors such as small IJV diameter or close proximity of the carotid artery.

Using VBUs as a radiographic reference for estimating catheter tip position offers several advantages.^3^ The thoracic spine is minimally affected by magnification and remains stable across postural changes.^4^ In our cohort, the mean carina-CAJ distance was 57.05±5.86 mm and correlated positively with patient height but not BMI. The correlation with height, but not with BMI, is physiologically plausible because thoracic skeletal dimensions are primarily determined by bone structure rather than by adiposity.

Mortality correlated weakly with age but not with ASA classification or metastatic disease. Because all ports were inserted on the right side in this study, future studies should compare bilateral approaches and incorporate multivariable modeling to better identify independent predictors of optimal catheter tip placement.

### Study Limitations

Several limitations should be acknowledged. First, the retrospective single-center design limits the generalizability of the findings. Second, although all patients were followed for at least six months, late catheter-related complications may have been underestimated. Third, the absence of multivariable analysis limits the identification of independent predictors of complications. Finally, the study population consisted solely of Turkish patients, which may further limit the external validity.

All catheterizations were performed on the right side, which limits extrapolation of the results to left-sided venous access. Therefore, caution is required when applying these findings to left internal jugular or SCV catheterizations.

## Conclusion

Our findings provide compelling evidence that ensuring the TIVAP catheter tip is positioned inferior to the carina and using the IJV as the preferred access route significantly reduce clinically relevant complications, particularly thromboembolism and catheter occlusion. Sub-carinal tip placement combined with IJV access should therefore be regarded as a best-practice strategy for safe TIVAP implantation.

Although LUM’s height-based measurement method offers a practical and equipment-free estimation tool, its moderate accuracy highlights the necessity of radiological confirmation of the the catheter-tip position. Future multicenter prospective studies with extended follow-up and comprehensive multivariable analysis are warranted to validate these findings.

**Supplementary Materials:** https://d2v96fxpocvxx.cloudfront.net/cf9d60d6-523c-458a-a2e6-78728d3ffbb0/content-images/378994b5-c1cd-4404-a7f1-6b11d71e371d.pdf

## Ethics

**Ethics Committee Approval:** This retrospective study was conducted after ethical approval by the University of Health Sciences Türkiye, Bağcılar Training and Research Hospital Ethics Committee (approval no.: 2023/10/09/065, date: 27.10.2023).

**Informed Consent:** Written informed consent was obtained from all patients prior to the procedure.

## Supplementary Materials

Supplementary Materials https://d2v96fxpocvxx.cloudfront.net/cf9d60d6-523c-458a-a2e6-78728d3ffbb0/content-images/378994b5-c1cd-4404-a7f1-6b11d71e371d.pdf

## Figures and Tables

**Table 1. Patient’s Demographics and Clinical Characteristics:** 

Age	Min-max (median)	23-82 (61)	
	Mean ± SD	59.2±11.8	
Gender	Male/n (%)	143 (48.2)	
	Female/n (%)	154 (51.9)	
Height (cm)	Min-max (median)	140-186 (165)	
	Mean ± SD	165.2±8.3	
Weight (kg)	Min-max (median)	42-108 (67)	
	Mean ± SD	68.9±12.7	
BMI (kg/m^2^)	Min-max (median)	15.8-54 (24.4)	
	Mean ± SD	25.3±4.8	
ASA score	Min-max (median)	0-4 (2)	
**Comorbidities**	**n (%)**		
- AKI	1 (0.3)	IHD	1 (0.3)
Arrhythmia	4 (1.4)	CAD	21(7)
Asthma	5 (1.7)	- HVD	2 (0.7)
- BPH	1 (0.3)	- CKD	2 (0.7)
- DM	74 (25)	- CHF	5 (1.7)
Hypo/hyperthyroidism	13 (4.4)	- COPD	19 (6.4)
- Hypotension	1 (0.3)	Obesity	2(0.7)
- HT	89 (39)	Tuberculosis	1 (0.3)
**Cancer type**	**n (%)**	-	
- Lung	3 (1)	Breast	42 (14.1)
- Brain	1 (0.3)	- Testicular	1 (0.3)
- Skin	2 (0.7)	- Tyroid	1 (0.3)
Hematological	1 (0.3)	- Uro-genital	7 (2.4)
Colorectal	131 (44.1)	Upper GI tract	102 (34.3)
- Larynx	6 (2)	-	
Metastasis	+/n (%)	56 (18.9)	
	−/n (%)	241 (81.1)	

Data presented as mean ± SD, Min-max (median) or count (%).

ASA, American Society of Anesthesiologists; AKI, acute kidney injury; BMI, body mass index; BPH, benign prostate hypertrophy; CAD, coronary artery disease; CHF, congestive heart failure; CKD, chronic kidney disease; COPD, chronic obstructive pulmonary disease; DM, diabetes mellitus; HT, hypertension; HVD, heart valve disease; IHD, ischemic heart disease; SD, standard deviation; Min-max, minimum-maximum

**Table 2. Effect of Catheterization Site on Complications and Patient Comfort Scale:** 

**-** **-**		**Internal jugular vein (n = 148)**	**Subclavian vein(n = 149)**	***P* value**
USG guidance	n (%)	144 (97.3)	20 (9.6)	<0.0001^a^
Number of venous puncture attempts	1 n (%)	104 (70.3)	94 (63.1)	0.1160^a^
	2 n (%)	31 (21)	30 (20.1)	
	3 and above n (%)	13 (8.8)	25 (16.8)	
Arterial puncture	n (%)	13 (8.8)	11 (7.4)	0.6577^a^
Pneumothorax	n (%)	2 (1.4)	2 (1.3)	0.9946^a^
Thromboembolism	n (%)	0 (0.00)	2 (1.3)	0.1573^a^
Catheter occlusion	n (%)	0 (0.00)	5 (3.4)	0.0246^a^
Local infection	n (%)	0 (0.00)	1 (0.7)	0.3181^a^
Patient comfort scale	Min-max (median)	0-3 (0)	0-5 (0)	0.1519^b^

^a^: *P* value was determined by chi-square test. ^b^: *P* value was determined by Mann-Whitney U test

USG, ultrasonography; Min-max, minimum-maximum

**Table 3. Imaging Measurement on CXR:** 

Catheter tip to CAJ (mm)	Min-max (median)	-44.80/110.20 (10.2)
	Mean ± SD	12.71±22.53
Carina to CAJ (mm)	Min-max (median)	41.70-72.70 (56.4)
	Mean ± SD	57.05±5.86
Position of the catheter tip relative to the carina	Below/n (%)	279 (93.9)
	Above/n (%)	18 (6.1)
Catheter tip in target zone	Yes/n (%)	178 (59.9)
	No/n (%)	119 (40.1)

Data presented as mean ± SD, min-max (median) or count (%)

CAJ, cavoatrial junction; CXR, chest radiography; SD, standard deviation; Min-max, minimum-maximum

**Table 4. The Effect of the Catheter tip Position Relative to the Carina on Postoperative Complications and the Number of Catheter Days:** 

-		**Below** **the carina** **(n=279)**	**Above** **the carina** **(n=18)**	***P* value**
Thromboembolism	n (%)	1 (0.4)	1 (5.6)	0.0090^a^
Catheter occlusion	n (%)	3 (1.1)	2 (11.1)	0.0013^a^
Number of catheter days	Min-max (median)	1-720 (200)	1-450 (180)	0.1243^b^
	Mean ± SD	236.60±149.80	175.40±110.70	

Data presented as mean ± SD, Min-max (median) or count (%). ^a^: *P* value was determined by chi-square test. ^b^:* P *value was determined by Mann-Whitney U test

SD, standard deviation; Min-max, minimum-maximum
